# Supplementary material for: Seminoma and Embryonal Carcinoma Footprints Identified by Analysis of Integrated Genome-Wide Epigenetic and Expression Profiles of Germ Cell Cancer Cell Lines
Source: PLoS One. 2014 Jun 2;9(6):e98330. doi: 10.1371/journal.pone.0098330 (PMC4041891; doi:10.1371/journal.pone.0098330)
Supplement: File S1 — ZIP file containing DMRforPairs output for significant regions. Please start from the html files. (ZIP) [file pone.0098330.s008.zip › figures/33153.pdf]

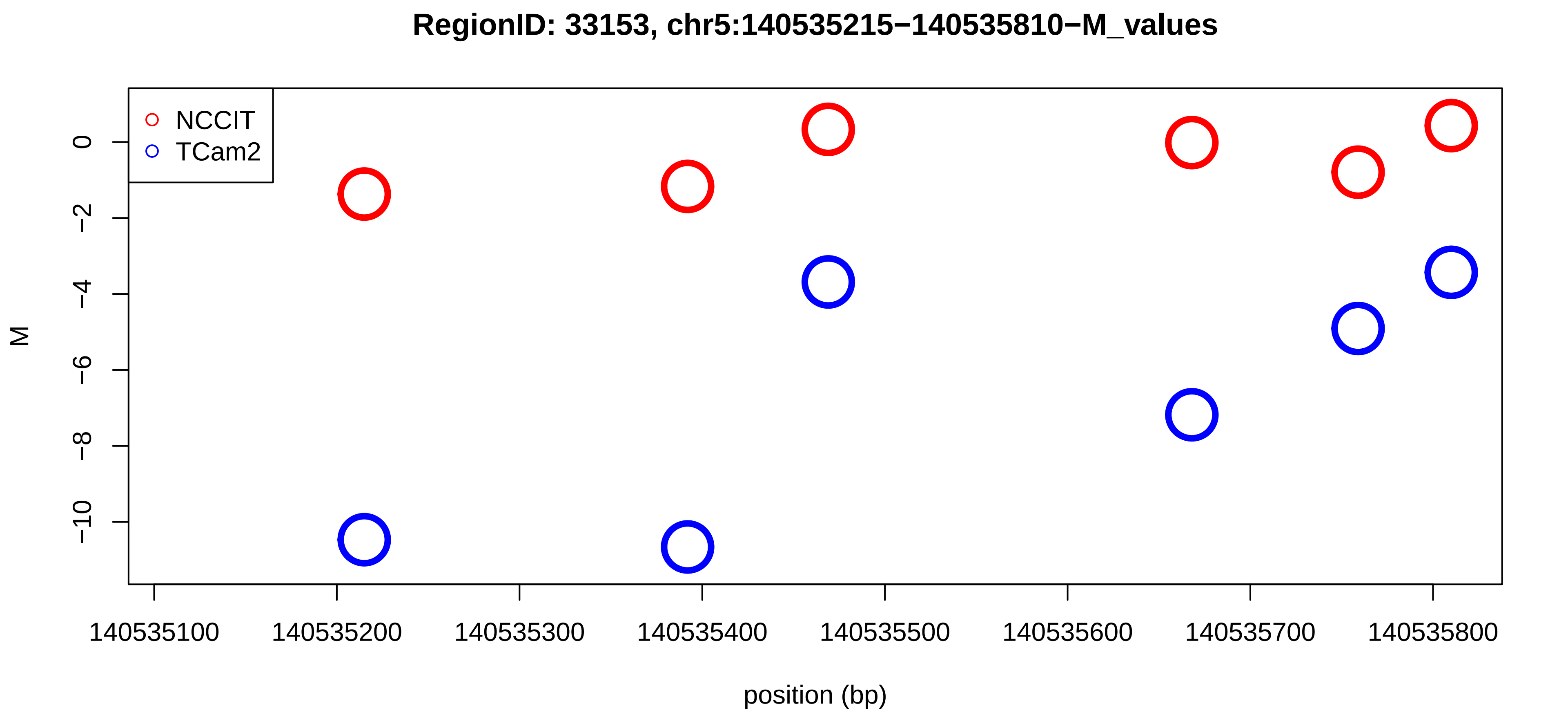

RegionID: 33153, chr5:140535215−140535810−M_values
NCCIT
TCam2
M
0
−2
−4
−6
−8
−10
140535100
140535200
140535300
140535400
140535500
140535600
140535700
140535800
position (bp)

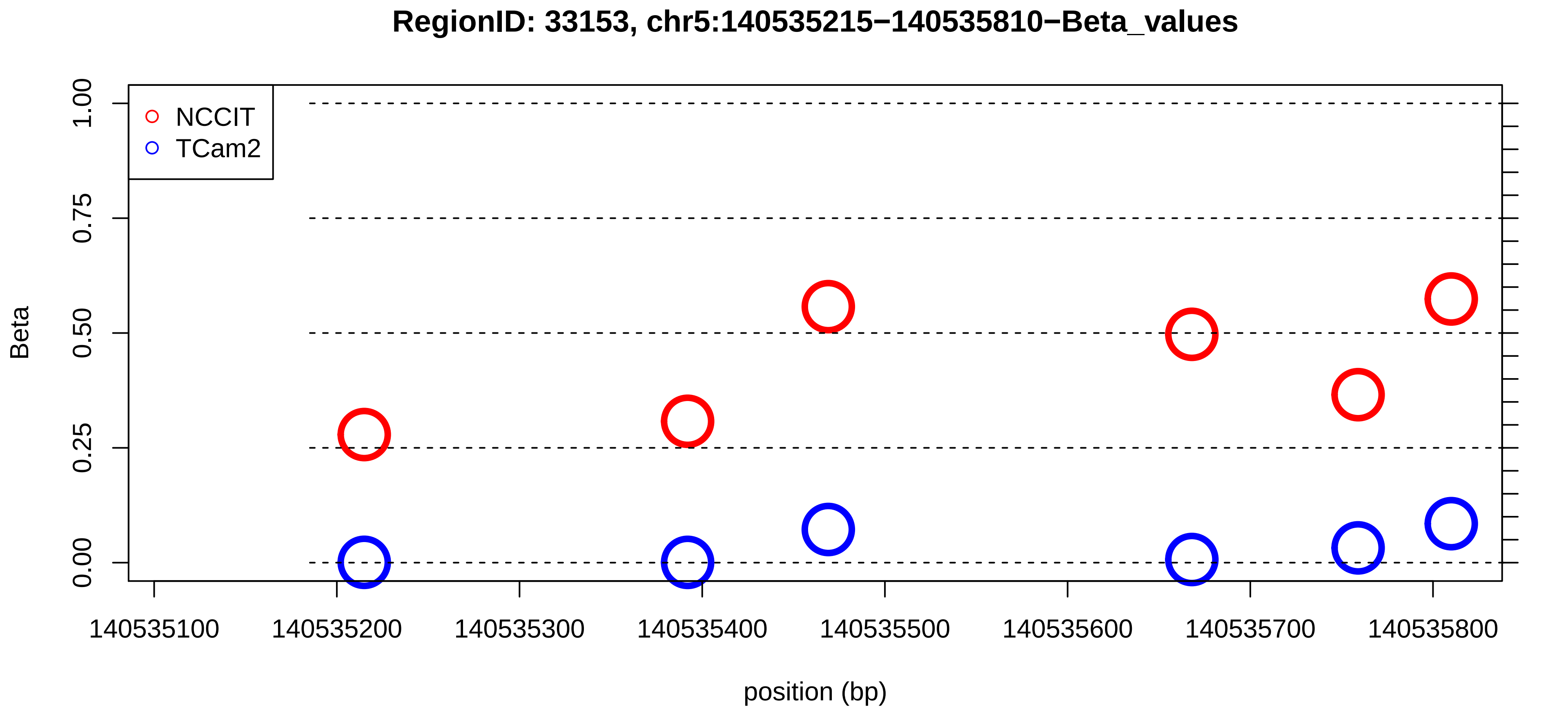

RegionID: 33153, chr5:140535215−140535810−Beta_values
NCCIT
TCam2
Beta
0.00
0.25
0.50
0.75
1.00
140535100
140535200
140535300
140535400
140535500
140535600
140535700
140535800
position (bp)
